# Effect of α-linolenic acid on endoplasmic reticulum stress-mediated apoptosis of palmitic acid lipotoxicity in primary rat hepatocytes

**DOI:** 10.1186/1476-511X-10-122

**Published:** 2011-07-25

**Authors:** Yong Zhang, Xia Yang, Hongyang Shi, Lei Dong, Jian Bai

**Affiliations:** 1 Department of Gastroenterology, the Second Affiliated Hospital of Xi'an Jiaotong University, No. 157, West 5th Road, Xi'an, Shaanxi Province-710004, China; 2 Anesthesia Department, the Second Affiliated Hospital of Xi'an Jiaotong University, No. 157, West 5th Road, Xi'an, Shaanxi Province-710004, China; 3 Department of Respiration, the Second Affiliated Hospital of Xi'an Jiaotong University, No. 157, West 5th Road, Xi'an, Shaanxi Province-710004, China; 4 Department of Clinical Medicine, Xi'an Medical University, No. 1, Xin Wang Road, Xi'an, Shaanxi Province-710021, China

## Abstract

**Background:**

Hepatic inflammation and degeneration induced by lipid depositions may be the major cause of nonalcoholic fatty liver disease (NAFLD). In this study, we investigated the effects of saturated and unsaturated fatty acids (FA) on apoptosis in primary rat hepatocytes.

**Methods:**

The primary rat hepatocytes were treated with palmitic acid and/or α-linolenic acid in vitro. The expression of proteins associated with endoplasmic reticulum (ER) stress, apoptosis, caspase-3 levels were detected after the treatment.

**Results:**

The treatment with palmitic acid produced a significant increase in cell death. The unfolded protein response (UPR)-associated genes CHOP, GRP78, and GRP94 were induced to higher expression levels by palmitic acid. Co-treatment with α-linolenic acid reversed the apoptotic effect and levels of all three indicators of ER stress exerted by palmitic acid. Tunicamycin, which induces ER stress produced similar effects to those obtained using palmitic acid; its effects were also reversed by α-linolenic acid.

**Conclusions:**

α-Linolenic acid may provide a useful strategy to avoid the lipotoxicity of dietary palmitic acid and nutrient overload accompanied with obesity and NAFLD.

## 1. Introduction

Nonalcoholic fatty liver disease (NAFLD) is a multifactorial disease [1,2] that illustrates a variety of symptoms, ranging from mild steatosis, nonalcoholic steatohepatitis to cirrhosis in the liver. Although it affects millions of people all over the world, the etiology of NAFLD is still unknown. However, hepatic inflammation and degeneration induced by the deposition of lipid droplets in the organ is considered as one of the major reasons of the disease [1-3]. In particular, certain saturated fatty acids (FA) such as palmitic acid can induce endoplasmic reticulum (ER) stress and apoptosis in rat and human liver cell lines [4-8], leading to inflammation and/or degeneration in the liver. This hypothesis is further demonstrated by the fact that ER stress and apoptosis could be induced by palmitic acid in both primary cells and cell lines derived from mice and rats [9,10].

Different fatty acids have different effects on ER stress. Palmitic acid is an effective inducer of ER stress [11][4]. Palmitic acid stimulates the synthesis of ceramides and increases reactive oxygen species [4][12], either of which may induce ER stress [12-14]. Palmitate modulates intracellular signaling, induces endoplasmic reticulum stress, and leads to apoptosis in mouse 3T3-L1 and rat primary preadipocytes [9].

We hypothesized that the cytoprotection provided by α-linolenic acid was a common function of rat primary hepatocytes and would be effective with clinically-relevant palmitic acid lipotoxicity. We have previously proved that α-linolenic acid protects against endoplasmic reticulum stress-mediated apoptosis of stearic acid lipotoxicity [15]. In this paper, we report that: (1) The characteristics of palmitic acid-mediated ER stress and apoptosis in primary hepatocytes; (2) α-linolenic acid could provide protection against the cell death induced by palmitic acid; (3) Take the role of GRP78, GRP94 expression and induction of CHOP into consideration, the beneficial effects were mediated via modification of the ER stress process with specific attention.

## 2. Materials and methods

### 2.1 Materials and cells

Rat hepatocytes were isolated from newborn (1-day-old) Sprague-Dawley rats using the modified two-step collagenase perfusion technique as previously described [16]. Freshly prepared hepatocytes were seeded at a density of 2 × 10^5 ^cells/well on 24-well multidishes precoated with rat tail collagen type I and grown in Williams Medium E containing 5% of fetal calf serum, 100 nM insulin, 2.5 μg/ml amphotericin B, 0.1 mg/ml gentamicin, 30 nM Na2SeO3, and 0.1 μM dexamethasone (Sigma-Aldrich, St. Louis, MO). Calf serum and amphotericin B were present for the first 24 h then omitted.

Cell culture materials and routine chemicals were obtained from Sigma (Oakville, ON, Canada) or Fischer Scientific (Nepean, ON, Canada). Primary antibodies were obtained from Stressgen (Victoria, ON, Canada) unless otherwise stated.

The experimental protocols were approved by the Animal Care and Protection Committee of Xi'an Jiaotong University.

### 2.2. Incubation of primary hepatocytes

Cultured hepatocytes at 80-90% confluence, were incubated with palmitic acid (250 μmol/l) for up to 16 h. Hepatocytes were also incubated with tunicamycin (5 μg/ml). Further incubations were also performed in which hepatocytes were incubated with palmitic acid (250 μmol/l) in the absence or presence of α-linolenic acid (150 or 250 μmol/l) for up to 16 h.

### 2.3. Measurement of cell viability and death

Cell viability and death were assessed as described previously by measurement of the enzymatic conversion of the yellow tetrazolium salt 3-(4, 5-dimethylthiazol-2-yl)-2, 5-diphenyltetrazolium bromide (MTT) into purple formazan and the release of lactate dehydrogenase (LDH) from lysed cells, respectively [17]. Primary rat hepatocytes were stained with Hoechst 33342-propidium iodide (HPI) to assess cell death by apoptosis and necrosis, respectively [18]. Specifically, apoptotic cells were distinguished as those with characteristic nuclear fragmentation and intense staining of condensed chromatin. Propidium iodide does not enter cells with intact plasma membranes, however, after entering damaged apoptotic or non-apoptotic cells it stains nuclear DNA pink. One thousand, randomly distributed nuclei were counted per sample and were scored as morphologically normal, apoptotic and necrotic using an inverted fluorescence microscope (Axiovert 25, Zeiss) set at excitation and emission wavelengths of 365 and 397 nm, respectively.

### 2.4. Measurement of caspase-3 activation

Caspase-3 activity was evaluated using a DEVD-NucViewTM 488 Caspase-3 substrate kit (Biotium Inc., Cambridge, UK). In the presence of active caspase-3 enzyme, the substrate dissociates from its bound fluorogenic DNA-binding dye and the latter binds to DNA and emits fluorescence. Caspase-3 was detected by microscopic examination and also by adapting the kit for microplate fluorescence reading. For this, cells were incubated with 50 μL of 5 μmol/L DEVD-NucView™ 488 Caspase-3 substrate for 30 min. Fluorescence was measured in a microplate reader (Cary Eclipse, Varian Inc.) set at wavelengths of 490 nm excitation and 520 nm emission.

### 2.5. Western blot analysis

Western blot analysis was performed as described in detail previously [19]. Membranes were incubated with antibodies against glucose-regulated protein 78 (GRP78; Stressgen, Victoria BC, Canada), glucose-regulated protein 94 (GRP94; Stressgen, Victoria BC, Canada), CCAAT/enhancer-binding protein homologous protein (CHOP; Santa Cruz Biotechnology, Santa Cruz, CA), and actin (Sigma). Proteins were detected with horseradish peroxidase-conjugated secondary antibodies (Amersham Pharmacia Biotech, Piscataway, NJ) and an enhanced chemiluminescence reagent (Pierce, Rockford, IL). Density was quantified using a UVP Bioimaging system (Upland, CA).

### 2.6. Statistical analysis

Results are expressed as mean ± standard error of the mean (S.E.M.) for n independent observations as indicated. Statistical differences between mean values of groups have been determined using one way analysis of variance (ANOVA) followed by a Dunnett's post-significance test for comparison of multiple means using the SPSS version 11.5. The level of significance was set at P < 0.05.

## 3 Results

### 3.1. Palmitic acid causes obvious cellular death of hepatocytes - protection by α-linolenic acid

**Figure 1:**
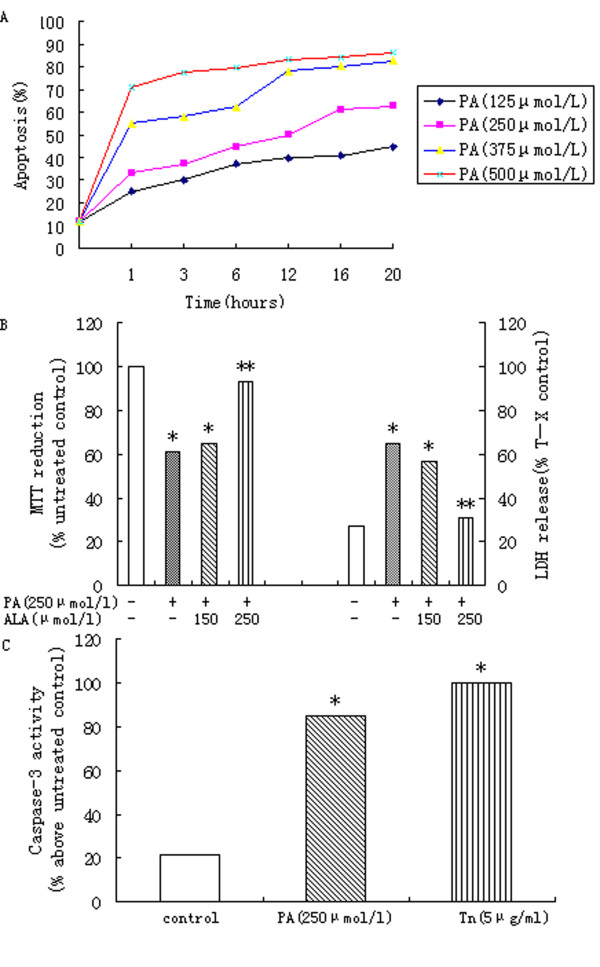
**Effects of PA on primary rat hepatocytes**. (A) Apoptotic cell death elicited by increasing concentrations of palmitic acid (PA). (B) Treatment with PA for 16 h produced significant and concentration-dependent effects on MTT reduction and LDH release. Data represent mean ± S.E.M., n = 5,*P < 0.05 vs. control (0 μmol/l palmitic acid), **P < 0.05 vs. palmitic acid-only cells. (C) Treatment of primary rat hepatocytes with 250 μmol/L PA produced a significant increase in activity of caspase-3. For comparison, the effects of tunicamycin(Tn; 5 μg/ml)are also shown. Data represent mean ± S.E.M., n = 5,*P < 0.05 vs. control (0 μmol/l palmitic acid), **P < 0.05 vs. palmitic acid-only cells.

Palmitic acid causes time and dose related apoptosis in primary rat hepatocytes (Figure 1A). Reduction of MTT and increased LDH release has showed incubation of subconfluent cultures of the hepatocytes with 250 μmol/l palmitic acid for 16 h produced a substantial loss of cell viability (Figure 1B). The mode of cell death observed at this concentration of palmitic acid was a combination of apoptosis and necrosis, which is observed using a combination of HPI staining (Figure 1B). However, co-incubation of primary rat hepatocytes with 250 μmol/l palmitic acid and 250 μmol/l α-linolenic acid promoted cell viability to levels observed in untreated cells (Figure 1B).

Palmitic-mediated apoptosis of primary rat hepatocytes coincided with a significant increase in caspase-3 activation (Figure 1C). Tunicamycin as well as palmitic acid increased fluorescence in the caspase-3 assay confirming activation of apoptosis pathways (Figure 1C).

### 3.2. α-Linolenic acid reduces ER stress mediated by palmitic acid

**Figure 2:**
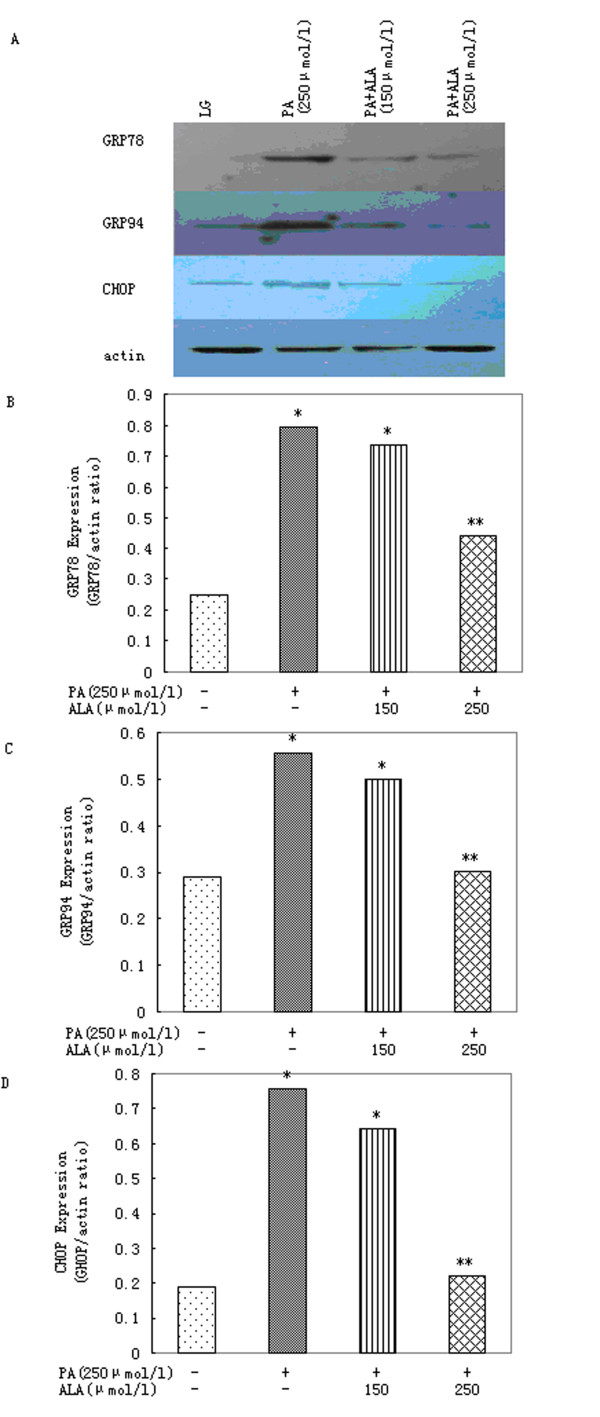
**α-Linolenic acid protects primary rat hepatocytes against ER stress induced by palmitic acid**. (A)Western blot and (B) densitometric analysis demonstrating the reduction of palmitic acid (PA)-induced GRP78 expression by 150 or 250 μmol/l α-linolenic acid (ALA) after 16 h. (A)Western blot and (C) densitometric analysis of GRP94 expression after 16 h incubation of cells with 250 μmol/l palmitic acid(PA) in presence of 150 or 250 μmol/l α-linolenic acid (ALA). (A)Western blot and (D) densitometric analysis demonstrating the reduction of palmitic acid (PA)-induced CHOP expression by 150 or 250 μmol/l α-linolenic acid (ALA) after 16 h. Data represent mean ± S.E.M., n = 5, *P < 0.05 vs. LG, low glucose control (0 μmol/l palmitic acid), **P < 0.05 vs. palmitic acid-only cells.

Palmitic acid produced a significant increase in the expression of markers of ER stress. After 16 h incubation with palmitic acid, increased levels of GRP78, GRP94 and CHOP were also detected (Figure 2).

In the presence of α-linolenic acid, ER stress mediated by palmitic acid was significantly reduced. Co-incubation of hepatocytes with 250 μmol/l palmitic acid and 250 μmol/l α-linolenic acid produced a significant reduction in levels of GRP78, GRP94 and CHOP after 16 h (Figure 2).

### 3.3. Effects of α-linolenic acid on primary rat hepatocytes death mediated by tunicamycin

**Figure 3:**
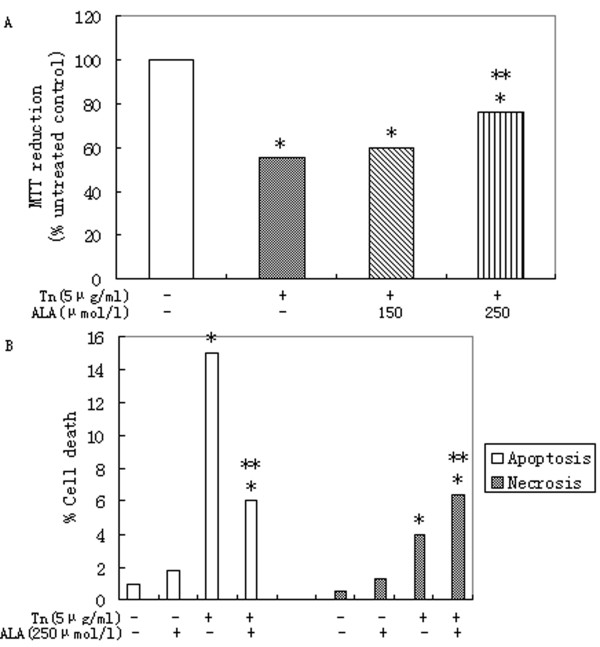
**α-Linolenic acid protects against dysfunction and apoptosis of primary rat hepatocytes induced by tunicamycin**. Relative cell death treated with 5 μg/ml tunicamycin (Tn) for 16 h in presence of 150 or 250 μmol/l α-linolenic acid (ALA). Data represent mean ± S.E.M., n = 5, *P < 0.05 vs. LG, low glucose control set to 1 (0 μmol/l palmitic acid), **P < 0.05 vs. tunicamycin-only cells.

Incubation of primary rat hepatocytes with 5 μg/ml tunicamycin for 16 h causes significant increases in cell death (Figure 3A). α-Linolenic acid at concentrations of 250 μmol/l was able to increase cell viability significantly (Figure 3A) which was confirmed by significant increases in both apoptosis and necrosis (Figure 3B). α-Linolenic acid at a concentration of 250 μmol/l increased significantly the necrotic cell death mediated by tunicamycin (Figure 3B).

### 3.4. Effects of α-linolenic acid on ER stress induced by tunicamycin

**Figure 4:**
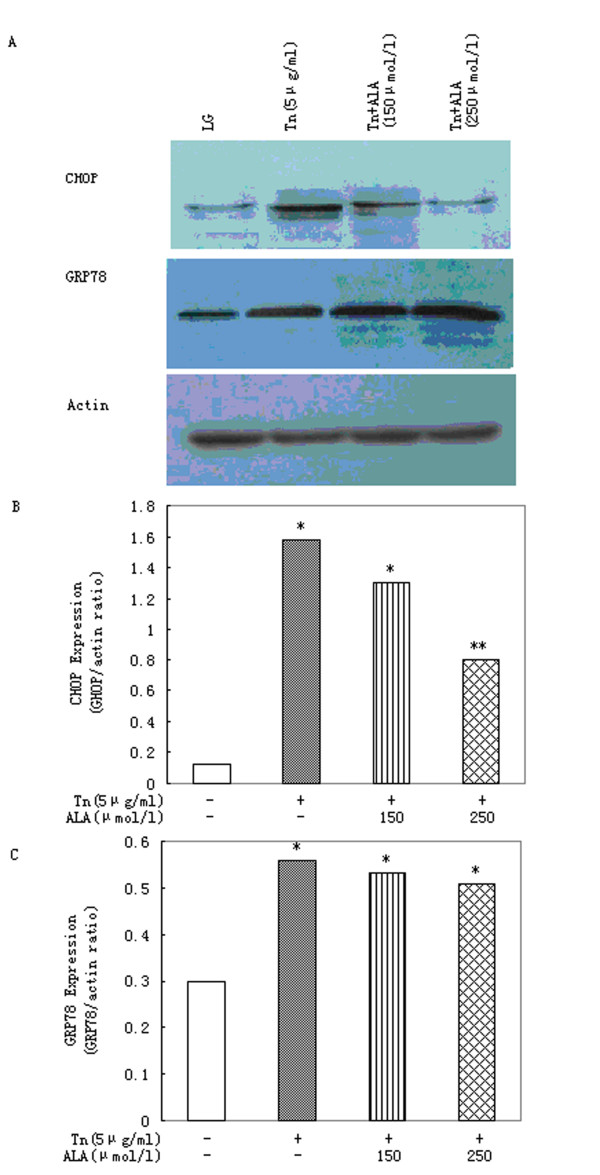
**α-Linolenic acid protects primary rat hepatocytes against ER stress induced by tunicamycin**. (A)Western blot image and densitometric analysis of (B) CHOP and (C) GRP78 expression in cells treated with tunicamycin (Tn; 5 μg/ml) in presence of increasing concentrations of α-linolenic acid (ALA) for 16 h. Data represent mean ± S.E.M., n = 5, *P < 0.05 vs. LG, low glucose control (0 μmol/l palmitic acid), **P < 0.05 vs. tunicamycin-only cells.

Incubation of primary rat hepatocytes with 5 μg/ml tunicamycin for 16 h produced a significant increase in levels of CHOP (Figure 4A, B). α-Linolenic acid, at concentrations of 250 μmol/l, was able to reduce the increase in CHOP levels produced by 5 μmol/l tunicamycin (Figure 4A, B). But, neither concentration was able to reduce GRP78 expression mediated by tunicamycin (Figure 4A, C).

## 4. Discussion

The present study was undertaken to elucidate the hypotheses that palmitic acid would induce ER stress and then cell death in liver cells, and that α-linolenic acid would inhibit these outcomes. Our study has three main findings. First, we have shown that the effects of a saturated and an unsaturated fatty acid, singly or in combination, upon induction of cell death, cell apoptosis, caspase-3 activity, ER stress in primary rat hepatocytes. Secondly, we have also illustrated the protein expression of three ER stress-associated genes in response to fatty acids treatment. The delivery and accumulation of lipids in non-adipose tissues leads to cellular dysfunction and death. This phenomenon, termed lipotoxicity, has been demonstrated in the pathogenesis of diabetes, cardiac failure and NAFLD [20-22]. Studies has found that damage of ER homeostasis and activation of the UPR in murine models of cardiac dysfunction, obesity and NAFLD [20,23,24].

Increased long chain saturated fatty acids induce ER stress, activate the UPR and promote cell death in a great amount of cell types, such as hepatocytes [25][21][4][26,27]. Thus, disruption in ER function seems lead to the pathogenesis of some diseases and to cellular impairments coupled with lipotoxicity. This was supported by our observations. Previously, we found that palmitic acid causes obvious cell death in primary rat hepatocytes. To elucidate the underlying mechanism of these effects, we found that the palmitic acid causes an obvious degree of ER stress in primary rat hepatocytes. Our results demonstrated that the ER stress response contributes to palmitic acid lipotoxicity. In addition, the studies showed that α-linolenic acid protects primary rat hepatocytes against palmitic acid lipotoxicity via reducing ER stress and apoptosis. Comparing with our previous study about stearic acid, we prove that 150 μmol/l α-linolenic acid provides very little benefits, which was presumed as the result of palmitic acid's stronger ability to elicited ER stress [15]. Furthermore, α-linolenic acid can reduce cellular dysfunction and apoptosis caused by tunicamycin. Tunicamycin, a well-known ER stress inducer, leads to apoptosis in a number of cells, including intestinal epithelial cells, renal cells and liver cells [28-30]. The mechanism on the basis of the cell necrosis induced by tunicamycin has not been clarified. These observations, together with our findings, suggest that α-linolenic acid protect primary rat hepatocytes through alleviation of tunicamycin-induced apoptosis.

The previous study sought to determine whether the ER stress and cell death in hepatocytes contributed to cell death in hepatocytes and to establish a link between these two [31]. Chop is among the best characterized of the UPR-regulated proapoptotic proteins [32]. Chop plays a role in disruption of ER homeostasis and apoptosis induced by long-chain saturated fatty acids. In several cell types, including liver, co-supplementation of oleate and palmitate reduces palmitate-mediated ER stress and apoptosis [4][33,34]. For this purpose, the ability of long chain saturated fatty acids to activate the ER-associated caspase-3 was examined.

Our work sheds new light on the mechanisms behind that ER stress produced by palmitic acid in primary rat hepatocytes can be considerably reduced by α-linolenic acid, an unsaturated fatty acid. We also manifest that an unsaturated fatty acid involves a reduction of ER stress which result in protection. Moreover, we examined the effects of a reduction in the raised levels of caspase-3 and CHOP coupled with palmitic acid. However, the results of our investigation seems to exclude a protective mechanism regulated by GRP78 as α-linolenic acid did not significantly affect levels of this chaperone molecule whereas levels of CHOP were substantialy reduced. In short, we concluded that α-linolenic acid may provide a useful strategy to avoid the lipotoxicity of dietary palmitic acid and nutrient overload accompanied with obesity and NAFLD.

## Competing interests

The authors declare that they have no competing interests.

## Authors' contributions

YZ conceived, designed and coordinated the work, as well as prepared the manuscript. LD was involved in the co-design of the work as well as the draft of the manuscript. XY, HS and JB carried out analytical work and contributed in drafting the manuscript. All authors read and approved the final manuscript.
